# Complete chloroplast genome sequence of *Solanum iopetalum*, one of the tuber-bearing wild potato relatives

**DOI:** 10.1080/23802359.2023.2183720

**Published:** 2023-02-28

**Authors:** Tae-Ho Park

**Affiliations:** Department of Horticulture, Daegu University, Gyeongsan, South Korea

**Keywords:** Chloroplast, genome, genome sequence, *Solanum iopetalum*

## Abstract

*Solanum iopetalum* belongs to the Solanaceae family and is one of the tuber-bearing wild *Solanum* species. In this study, chloroplast genome sequencing of the species, completed with Illumina sequencing technology, is presented. The length of the chloroplast genome is 155,625 bp with a GC content of 37.86%. It comprises a large single copy (LSC) region of 86,057 bp, a small single copy (SSC) region of 18,382 bp, and two inverted repeat regions (IRa and IRb) of 25,593 bp. Additionally, 158 functional genes in the genome are identified, including 105 protein-coding genes, 8 ribosomal RNAs, and 45 transfer RNAs. Phylogenetic analysis revealed that *S. iopetalum* is grouped into a large clade with other *Solanum* species, including cultivated potatoes (*S. tuberosum*) and is closely related to Mexican *Solanum* species (*S. stoloniferum*, *S. verrucosum*, *S. hougasii*, *S. hjertingii*, and *S. demissum*). This study provides useful genomic information for future breeding and evolutionary studies of *S. iopetalum* and other *Solanum* species.

## Introduction

*Solanum iopetalum* (Bitter) Hawkes 1944, which is endemic to Mexico, is one of the tuber-bearing wild relatives of cultivated potatoes (*Solanum tuberosum*) (Hawkes 1990). Due to its resistance to *Phytophthora infestans* (Pacheco-Sánchez et al. 2003; Tiwari et al. 2015) and its resistance to several abiotic stresses such as drought, heat, and salt (data identified in contemporary research not shown), it could be one of the great resources for potato breeding. Its endosperm balance number (EBN) of four is the same as that of *S. tuberosum*, which theoretically allows direct cross between the two different species for potato breeding (Hawkes 1990; Ortiz and Ehlenfeldt 1992; Cho et al. 1997). However, the different multiple ploidy levels causing an inconvenience in breeding must be overcome, because *S. iopetalum* is hexaploid and *S. tuberosum* is tetraploid (Hanneman 1994). Moreover, it has been considered an allohexaploid based on facts proposed by Hawkes (1990). *S. iopetalum* might be produced as a hybridization of the *Rotata* species migrated from South America to Mexico and the Central American *stellate* diploids (Hawkes 1990). Its nuclear genome composition has been identified as allohexaploid with two (A and P) or three (A, B, and P) component genomes from diploid North American (A only, or A and B) and South American (P) species (Spooner et al. 2004; Pendinen et al. 2012). However, the results obtained from analyses of rDNA repeats and 5S rDNA suggest that *S. iopetalum* could be created by hybridization of closely related species, or it is autopolypoid (Volkov et al. 2001, 2003). Therefore, the origin of the Central American polyploid *Solanum* species *S. iopetalum* is still unclear, and the plastid genome of the species has rarely been studied.

## Materials and methods

**Figure 1.**
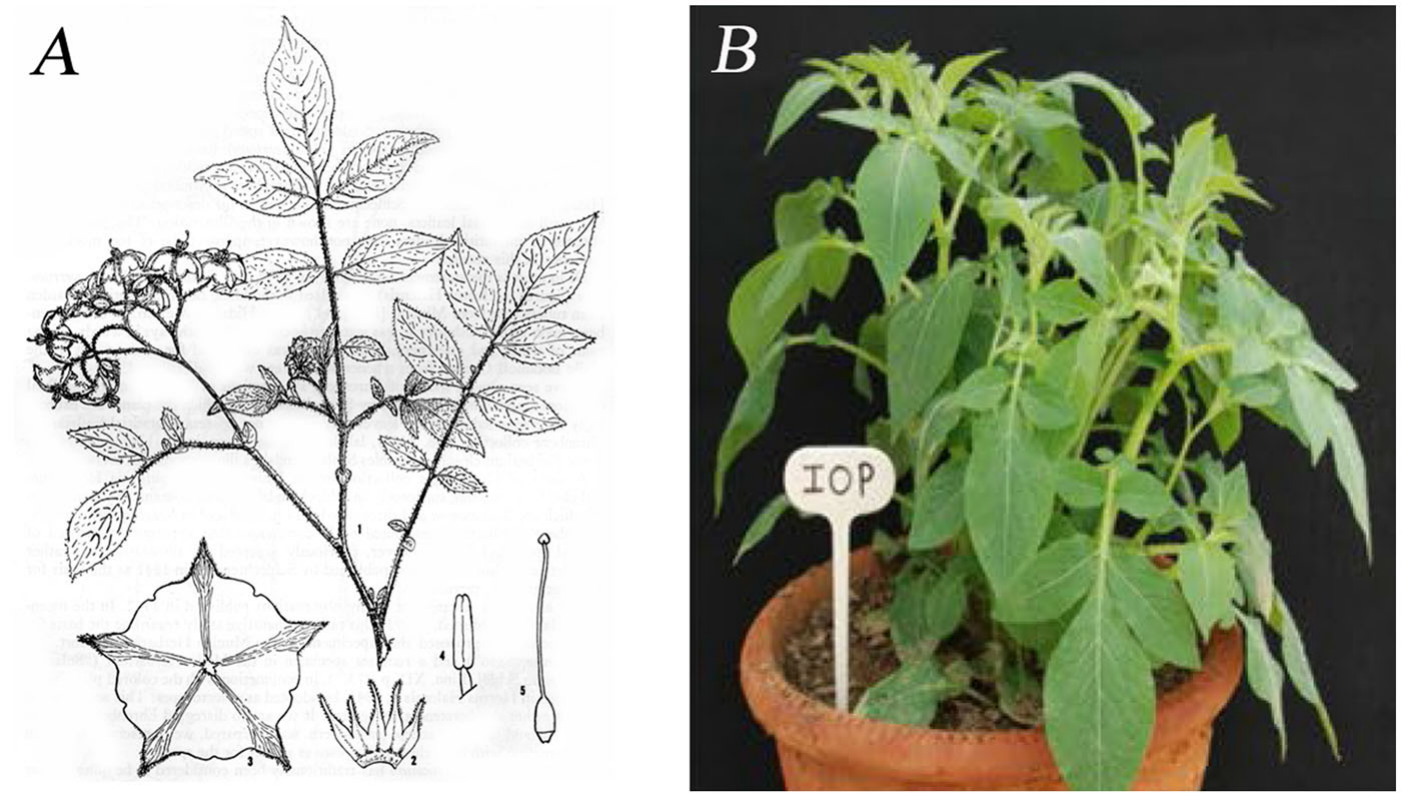
The species reference images for *Solanum iopetalum*. (A) Illustration of *S. iopetalum* (Correll 1962). (B) Plant shape of *S. iopetalum* (Tiwari et al. 2019). IOP is the three letter abbreviation for *S. iopetalum.*

*Solanum iopetalum* plants (PI230459) were provided by Highland Agriculture Research Institute, South Korea (37°68′05.4″N 128°73′09.1″E) (Figure 1), and the specimen was deposited in the National Agrobiodiversity Center, South Korea (http://genebank.rda.go.kr/, Young-Eun Park, papalove@korea.kr) under voucher number IT301490.

Total genomic DNA was extracted from the leaves of the *S. iopetalum* plants using the Genomic DNA Extraction Kit for plants (RBC Bioscience, New Taipei City, Taiwan) according to the manufacturer’s instructions. For chloroplast genome sequencing of the species, basically performed via Phyzen bioinformatics pipeline (Kim et al. 2015), the library was constructed based on the PE standard protocol (Illumina, San Diego, USA). Paired-end (PE) sequencing was performed on the Illumina HiSeq2000 platform. The clean reads were assembled with the dnaLCW method using the CLC *de novo* assembly program in CLC assembly cell package version 4.2.1 (CLC Inc, Rarhus, Denmark). The structure and sequence of the assembled chloroplast genome were analyzed with the results of a BLASTN search at the NCBI database and BLASTZ analysis (Schwartz et al. 2003) using the *S. gourlayi* complete chloroplast genome (GenBank accession no. MH021474) as a reference. Chloroplast genome annotation was performed by using the GeSeq program (Tillich et al. 2017) and the circular genome map was generated using the OGDraw program (Lohse et al. 2007). The schematic map of the trans- and cis-splicing genes were generated using the CPGview software (Liu et al. 2023, http://www.1kmpg.cn/cpgview/).

## Results and discussion

**Figure 2.**
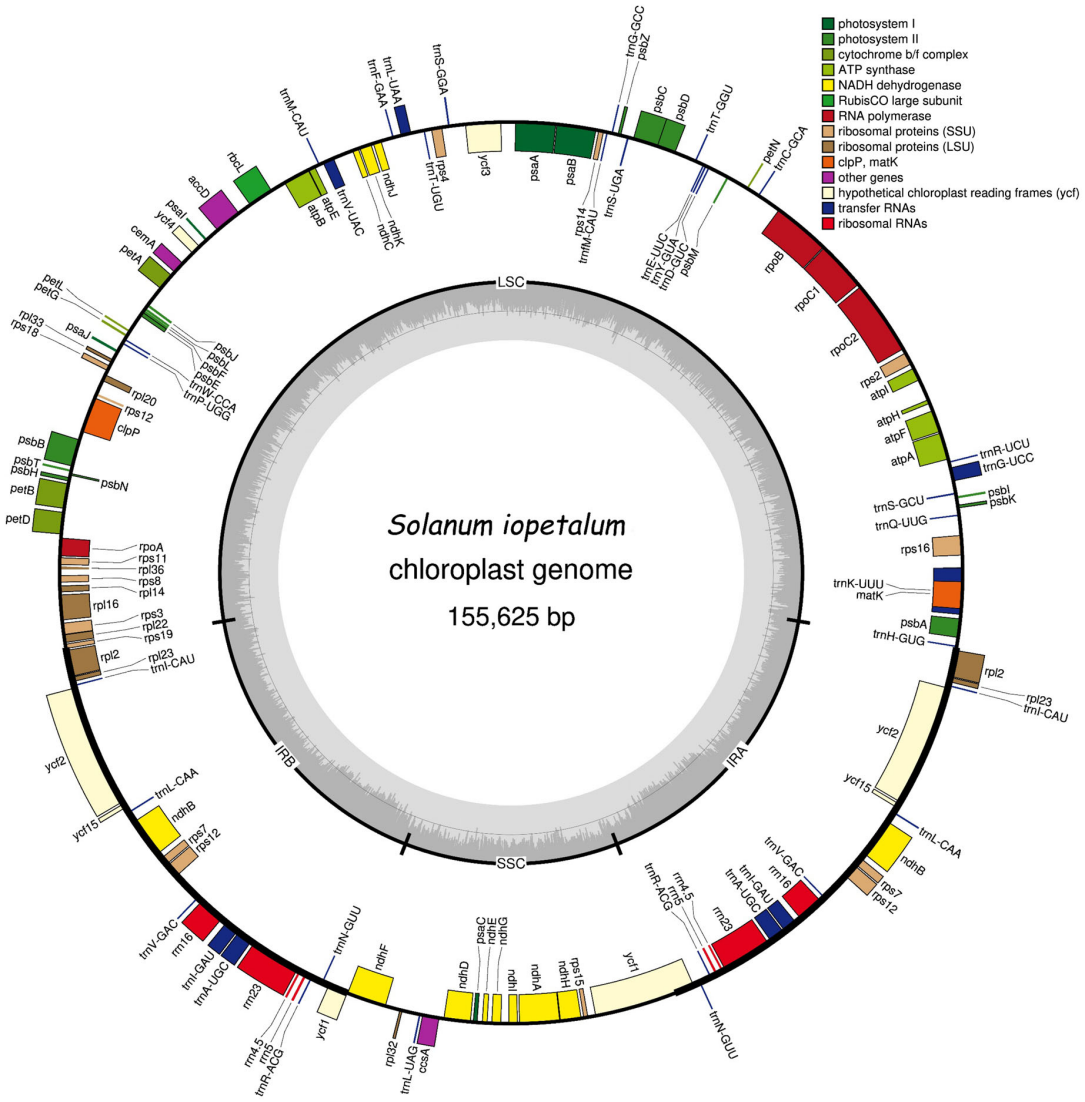
Gene map of the *Solanum iopetalum* chloroplast genome. Genes on the outside and inside of the map are transcribed in clockwise and counterclockwise directions, respectively.

In total, approximately 2.84 Gbp of raw data were obtained and approximately 2.22 Gbp of the clean reads were assembled. The average coverage is 405.61 and mapped read depth through the whole region is approximately at least more than 200 (Supplementary Figure 1). The whole chloroplast genome sequence of *S. iopetalum* is 155,625 bp in length with a typical double-stranded loop structure. It is divided into four regions consisting of a large single copy (LSC) region of 86,057 bp, a small single copy (SSC) region of 18,382 bp, and two inverted repeat regions (IRa and IRb) of 25,593 bp. The overall GC content was 37.86%. The BLASTN search results showed that the sequence of *S. iopetalum* is most similar with that of *S. gourlayi*, which also originates from Mexico. The similarity between these species was also observed for the External Transcribed Spacer (ETS) sequence (Volkov et al. 2003) and the 5S rDNA sequence (Volkov et al. 2001). The *S. iopetalum* chloroplast genome contains a total of 158 genes with an average size of 583.1 bp, including 105 protein-coding genes, 45 transfer RNA genes, and eight ribosomal RNA genes, with average sizes of 764.7, 62.0, and 1,131.0 bp, respectively (Figure 2). Eleven protein coding genes, 9 tRNA genes, and 4 rRNA genes are duplicated in the IR regions. The *rps12* is a trans-splicing gene (Supplementary Figure 2A) and thirteen genes including *rps16*, *atpF*, *rpoC1*, *ycf3*, *clpP*, *petB*, *petD*, *rpl16*, *rpl2*, *ndhB*, *ndhA*, *ndhB*, and *rpl2* are cis-splicing genes (Supplementary Figure 2B). Gene features are typically identical to those of higher plants. The results of chloroplast genome assembly and annotation were submitted to GenBank (http://www.ncbi.nlm.nih.gov/) under accession number MZ233587.

**Figure 3.**
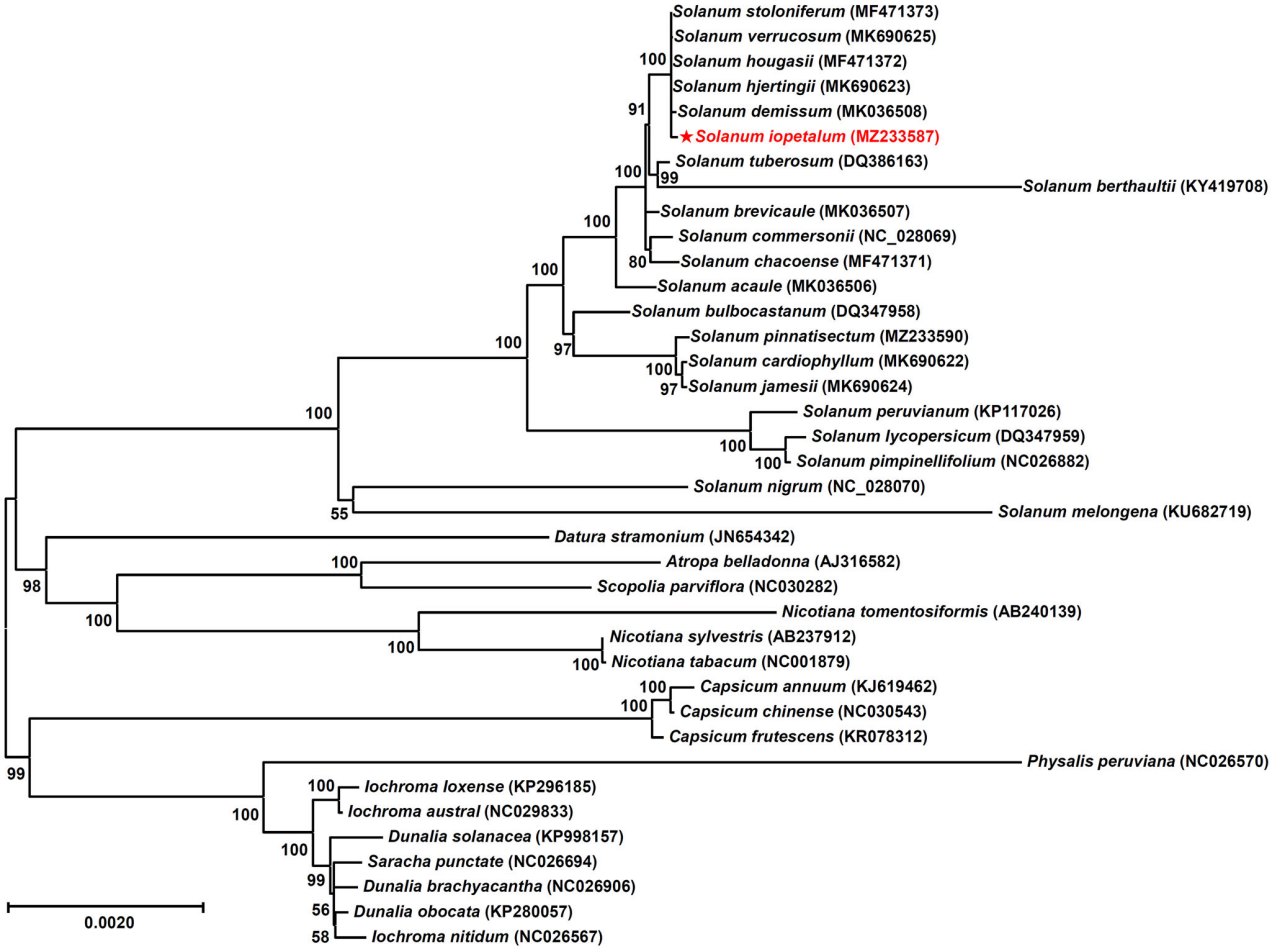
Maximum likelihood phylogenetic tree of *Solanum iopetalum* with other species belonging to the Solanaceae family based on chloroplast protein coding sequences. Numbers in the nodes are the bootstrap values from 1,000 replicates. The data have been partially adopted from Park (2022a). The following 38 sequences were used: MF471373 (Park 2018), MK690625, MF471372 (Cho et al. 2018), MK690623 (Park 2022c), MK036508 (Cho et al. 2019), MZ233587 (this study), DQ386163 (Gargano et al. 2012), KY419708 (Park 2017), MK036507 (Park 2019), NC028069 (Cho et al. 2016), MF471371 (Cho et al. 2017), MK036506 (Park 2021), DQ347958 (Daniell et al. 2006), MZ233590, MK690622, MK690624, KP117026 (Wu 2016), DQ347959 (Daniell et al. 2006), NC026882 (Wu 2016), NC028070 (Park 2016), KU682719 (Ding et al. 2016), JN654342, AJ316582 (Schmitz-Linneweber et al. 2002), NC030282 (Park and Lee 2016), AB240139 (Yukawa et al. 2006), AB237912 (Yukawa et al. 2006), NC001879 (Shinozaki et al. 1986), KJ619462 (Zeng et al. 2016), NC030543 (Park et al. 2016), KR078312 (Shim et al. 2016), NC026570, KP296185, NC029833, KP998157, NC026694, NC026906, KP280057, NC026567.

To determine the phylogenetic status of *S. iopetalum*, 37 other species in the Solanaceae family were obtained from the GenBank database. The phylogenetic tree was constructed using a maximum likelihood method with a Kimura 2-parameter model based on chloroplast coding sequences. The analysis was conducted using MEGA 6.0 with 1,000 bootstrapping options (Tamura et al. 2013). The results showed that *S. iopetalum* is clustered with other species in the genus *Solanum* and is closely related to *S. stoloniferum*, *S. verrucosum*, *S. hougasii*, *S. hjertingii*, and *S. demissum* in the cluster (Figure 3). These five species are also *Solanum* members from Mexican although they have different ploidy levels and EBNs. Spooner and Sytsma (1992) and Spooner et al. (1995, 2008) reported similar results from data with chloroplast DNA restriction site variations, morphological characteristics, and cloned DNA sequences of the single-copy nuclear gene Granule-Bound Starch Synthase I (GBSSI or waxy).

## Conclusion

The chloroplast genome of *S. iopetalum* was characterized for the first time in this study. The plastome length of *S. iopetalum* is comparable to other *Solanum* species, but slightly longer than others (Park 2022b). Our findings will facilitate further research investigating more detailed breeding and evolutionary aspects.

## Ethical approval

This study did not involve endangered or protected species, and the plant was collected with the permission from Highland Agriculture Research Institute (37^°^68′05.4"N 128^°^73′09.1"E), Pyeongchang, South Korea.

## Supplementary Material

Supplemental Material

Supplemental Material

## Data Availability

The genome sequence data that support the findings of this study are openly available from NCBI (https://www.ncbi.nlm.nih.gov/) under accession number MZ233587. The associated BioProject, SRA, and BioSample numbers are PRJNA729825, SRR14532939, and SAMN19180224, respectively.
