# Supplementary figures and images for: Complete chloroplast genome sequence of Solanum iopetalum, one of the tuber-bearing wild potato relatives
Source: Mitochondrial DNA B Resour. 2023 Feb 28;8(3):347–51. doi: 10.1080/23802359.2023.2183720 (PMC9980020; doi:10.1080/23802359.2023.2183720)

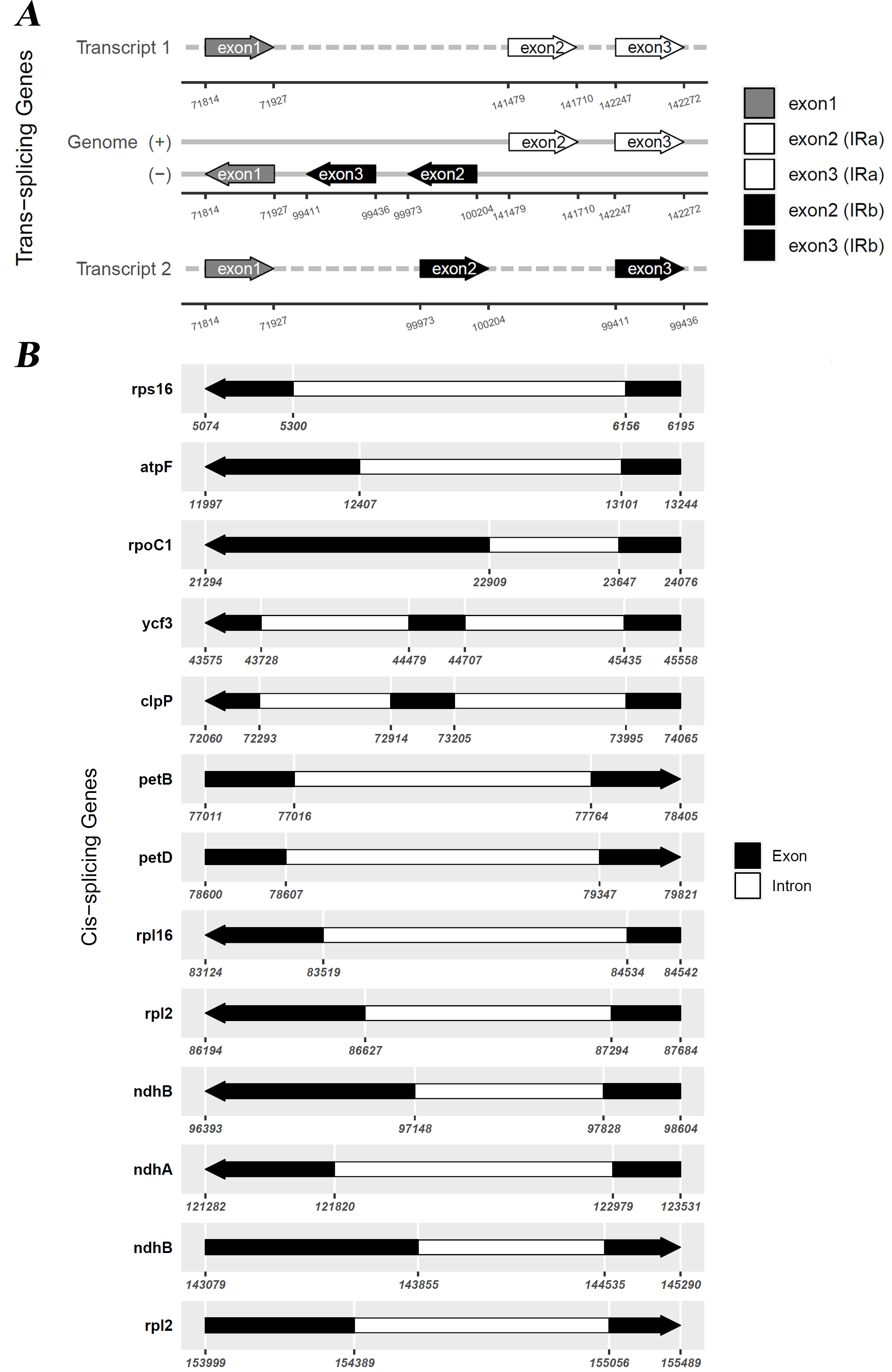

Supplement: Supplemental Material [file TMDN_A_2183720_SM4197.jpg]

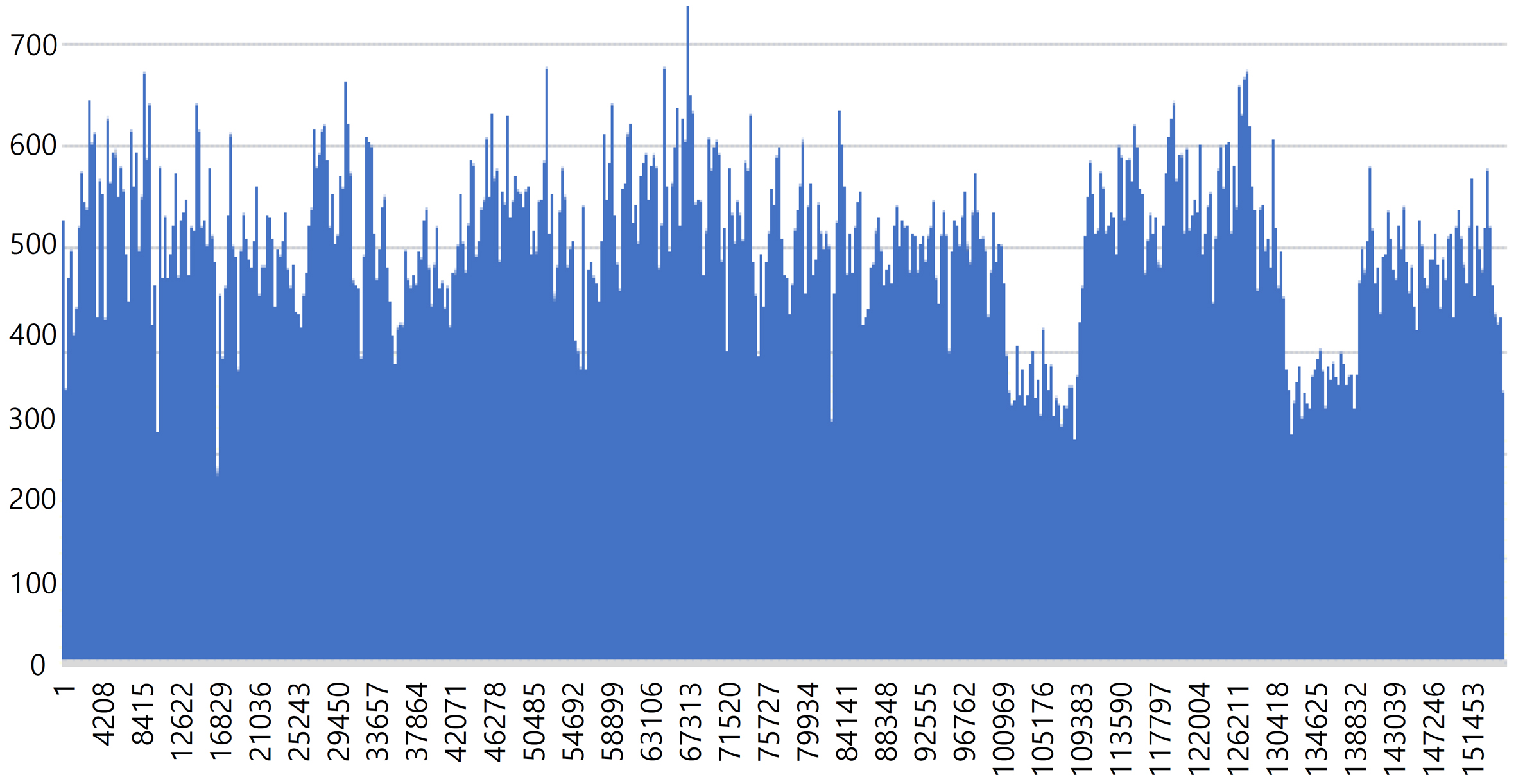

Supplement: Supplemental Material [file TMDN_A_2183720_SM4162.jpg]
